# Intelligent Membranes: Dream or Reality?

**DOI:** 10.3390/membranes3030151

**Published:** 2013-07-15

**Authors:** Annarosa Gugliuzza

**Affiliations:** Institute on Membrane Technology—Research National Council (ITM-CNR), c/o University of Calabria, Via P. Bucci, 17C, 87036 Rende (CS), Italy; E-Mails: a.gugliuzza@itm.cnr.it; annarosa.gugliuzza@cnr.it; Tel.: +39-(0)984-492026; Fax: +39-(0)984-402103

**Keywords:** responsive membranes, sensors, biomimetic interfaces, wetting, transport, separation, self-cleaning, self-healing, electronic textiles, smart gels, actuators

## Abstract

Intelligent materials are claimed to overcome current drawbacks associated with the attainment of high standards of life, health, security and defense. Membrane-based sensors represent a category of smart systems capable of providing a large number of benefits to different markets of textiles, biomedicine, environment, chemistry, agriculture, architecture, transport and energy. Intelligent membranes can be characterized by superior sensitivity, broader dynamic range and highly sophisticated mechanisms of autorecovery. These prerogatives are regarded as the result of multi-compartment arrays, where complementary functions can be accommodated and well-integrated. Based on the mechanism of “sense to act”, stimuli-responsive membranes adapt themselves to surrounding environments, producing desired effects such as smart regulation of transport, wetting, transcription, hydrodynamics, separation, and chemical or energy conversion. Hopefully, the design of new smart devices easier to manufacture and assemble can be realized through the integration of sensing membranes with wireless networks, looking at the ambitious challenge to establish long-distance communications. Thus, the transfer of signals to collecting systems could allow continuous and real-time monitoring of data, events and/or processes.

Intelligent membranes are considered part of the category of the ultra-smart systems, which are regarded as living structures able to sense, adapt themselves and act in response to physical or chemical changes occurring in the surrounding environment. Under the effect of one or more external stimuli—*light, temperature, pH, ionic strength, affinity, solvent, electrical and magnetic fields, etc.*—intelligent membranes can self-adjust their structural and chemical features in order to regulate mass and heat flow, but also to store and provide energy, detect and protect against harmful agents/irradiations, switch wetting properties, transfer information/signals, and so many other procedures [1].

Based on the “sensing-to-action” sequence, stimuli-responsive membranes can offer attractive solutions to numerous applications in the field of textiles, biomedicine, environment, agro-food, water treatment, agriculture, architecture, transport and energy [2,3,4].

Conceptually, the membrane is considered as a semi-permeable interface able to block the passage of some species while other substances are transported from one phase to another, under steady or unsteady conditions. The transport can be controlled through different mechanisms depending on the membrane feature-types, stream chemistry, and process engineering [5,6,7,8]. 

Unquestionably, in the last years the membrane science has gained a leadership in traditional areas such as separation, purification, chemical and energy conversion, by-products reduction, development of artificial organs, and so on [9].

However, it is quite surprising how the way of perceiving the concept of membrane has been progressively changed, leading to an emerging and always more expanding area of research, wherein membrane and sensor technologies are vigorously examined in an attempt to mimic the behavior of natural systems, which are regarded as “multi-reservoirs of intricate and miniaturized smart functional architectures” [10,11,12,13,14]. 

In this frame, many efforts have been done so far in order to make the membranes adaptable, self-maintaining and self-healing like objects, animals and vegetables broadly distributed in nature. Thus, it is not difficult to imagine how transport, wetting, transcription, hydrodynamics, and chemical or energy conversion can be triggered through artificial membranes having a flat, tubular or spherical configuration [15,16,17,18,19].

A major success is realized when the control of the matter occurs on nano and microscale [20,21,22]. Indeed, various bottom-up and top-down approaches have been extensively thought out to combine components with complementary functions in predetermined volumetric spaces, leading to complex multifunctional systems, where desired functions associated with tailored structures can be solicited when sought [23]. 

Undoubtedly, the creation of interactive and ultra-smart membranes will bring substantial changes in technology, economy, lifestyle and society. It is enough to imagine the advantages arising from the use of intelligent membranes in architecture, agriculture, textiles, pharmaceutical, food and beverage industries. The ability of membranes to stop the air permeability and change the water transmission rate, depending on the direction of heat flow and relative humidity, is expected to promote the development of smart and energy-saving constructions over seasonal time, but also to make textiles comfortable and adaptable to extreme activities and environmental conditions. The smart regulation of vapors and gases, including CO_2_ and O_2_, is surely an attractive challenge for application in packaging, where prolonged freshness, quality monitoring, safety and harmless shipping/storage represent important prerogatives.

Biotechnology is another area where intelligent membranes are in great demand for human health, defense and security. Stimuli-responsive membranes, working as reservoirs of target molecules for targeted therapies or against harmful agents, represent the focus of many current membrane-based studies as well as membranes working like chemical valves and of interest for selective bio-separations, ion-transport and molecular capture. Membranes capable of detecting, stopping and/or quickly destroying contaminants and dangerous chemical or biological compounds could come into play in military, aerospace, biomedicine, water treatment or environmentally hazardous compartments as well.

Also, smart membranes perform a fascinating function when used for self-cleaning and self-healing purposes. Combining morphological features with sensing chemical elements, it is possible to save original properties and integrity of the membranes, taking advantage of preventing fouling phenomena, dirty/soil adhesion or damage in the structure and/or chemistry. 

Optimistically, the future is the miniaturization of adaptive materials and nanosensors in membranes with a major ambition to connect the latter to wireless networks in order to transfer/store signals and data, establishing long-distance communications. 

It is evident that the achievement of this target could fully revolutionize the lifestyle of everyone and especially that of sportsmen, astronauts, babies, soldiers and the sick. The integration of conductive wearing membranes in textiles appears to be, for example, a viable route towards the fabrication of comfortable electronic devices, enabling to detect in real-time vital functions such as breathing, cardiac frequency, sweat, temperature, blood, and posture [24].

Future prospects involve highly motivated membrane technologists working on the creation of membranes such as complex arrays where new properties can be accommodated for, giving varied smart responses to direct inputs arising from the surrounding environment and, therefore, provide benefits without sacrificing performance. 

Then, the dream may come true ….
